# Chimeric Antigen Receptor-T Cells in Colorectal Cancer: Pioneering New Avenues in Solid Tumor Immunotherapy

**DOI:** 10.1200/JCO-24-02081

**Published:** 2025-01-13

**Authors:** Shaida Ouladan, Elias Orouji

**Affiliations:** ^1^ Department of Pathology, McGill University, Montreal, QC, Canada; ^2^ Princess Margaret Cancer Centre, University Health Network, Toronto, ON, Canada

## Abstract

Colorectal cancer (CRC) remains a major global health burden, being one of the most prevalent cancers with high mortality rates. Despite advances in conventional treatment modalities, patients with metastatic CRC often face limited options and poor outcomes. Chimeric antigen receptor-T (CAR-T) cell therapy, initially successful in hematologic malignancies, presents a promising avenue for treating solid tumors, including CRC. This review explores the potential of CAR-T cell therapy in CRC by analyzing clinical trials and highlighting prominent CRC-specific targets. We discuss the challenges such as immunosuppressive microenvironment, tumor heterogeneity, and physical barriers that limit CAR-T efficacy. Emerging strategies, such as logic-gated and dual-targeting CAR-T cells, offer practical solutions to overcome these hurdles. Furthermore, we explore the combination of CAR-T cell therapy with immune checkpoint inhibitors to enhance T-cell persistence and tumor infiltration. As the field continues to evolve, CAR-T cell therapies hold significant potential for revolutionizing the treatment landscape of CRC.

## INTRODUCTION

Colorectal cancer (CRC) is a significant global health challenge, being the third most common cancer and the second leading cause of cancer-related deaths worldwide.^1,2^ Traditional treatment modalities such as surgery, chemotherapy, and radiation therapy, although effective in many cases, often fall short for patients with advanced or metastatic CRC.^3^ In recent years, immunotherapy has emerged as a promising avenue, with chimeric antigen receptor-T (CAR-T) cell therapy being one of these innovative treatments.^4,5^

**FIG 1.**
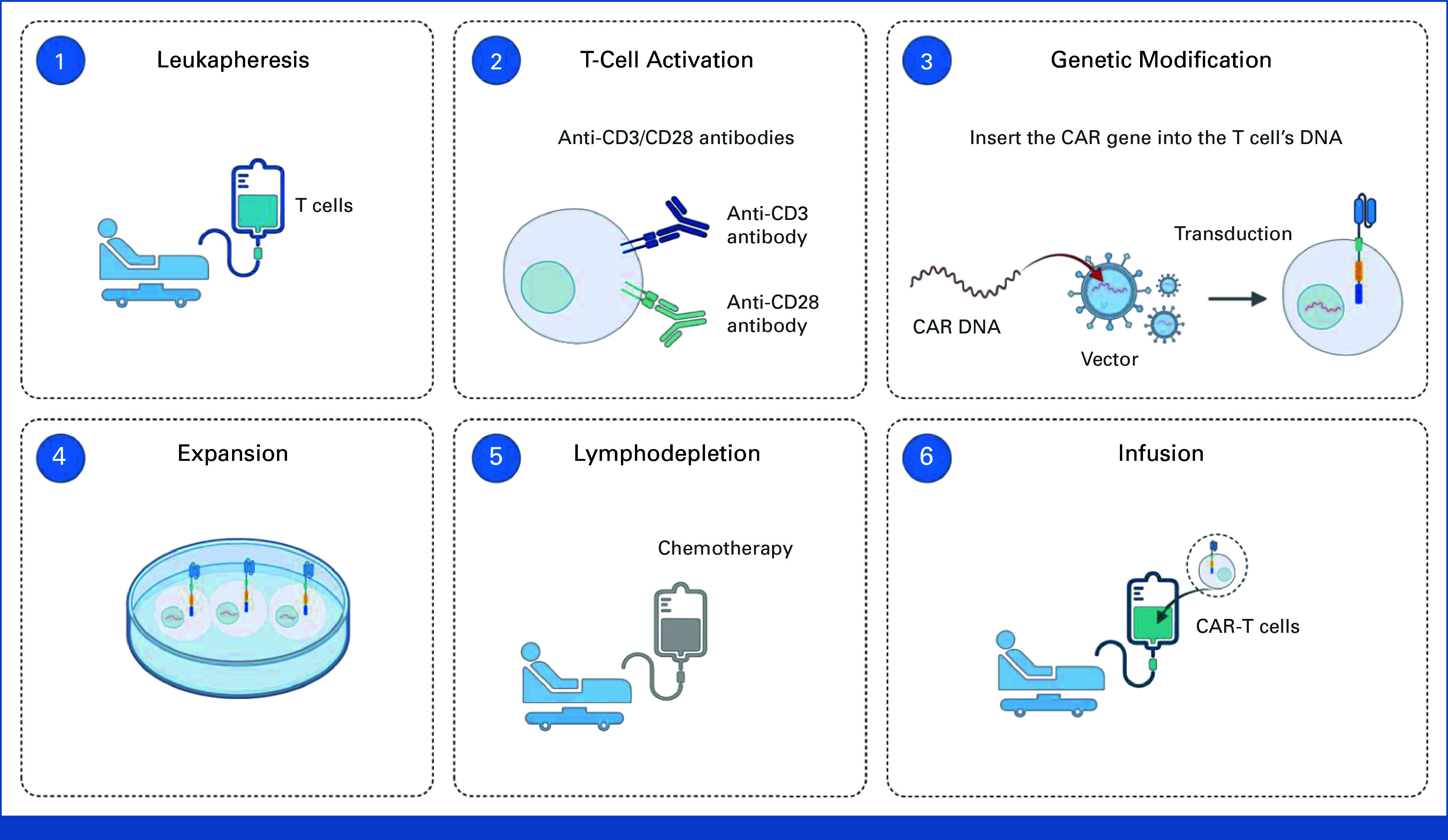
Overview of the CAR-T cell therapy process, highlighting the sequential steps from leukapheresis to CAR-T cell infusion. (1) T cells are isolated from the patient's peripheral blood through leukapheresis. (2) These cells are activated using anti-CD3/CD28 antibodies to stimulate their proliferation. (3) Genetic modification of the T cells is performed by inserting the CAR gene, enabling them to recognize tumor-specific antigens. (4) CAR-T cells are expanded ex vivo to achieve sufficient quantities for therapeutic use. (5) Before reinfusion, the patient undergoes lymphodepletion, typically via chemotherapy, to enhance CAR-T cell engraftment. (6) CAR-T cells are infused back into the patient, where they target and eliminate cancer cells expressing the CAR-specific antigen. CAR-T, chimeric antigen receptor-T.

CAR-T cell therapy genetically modifies a patient's T cells to target cancer cells by inserting a CAR.^6,7^ The process involves collecting T cells, activating them, genetically modifying them with a CAR to recognize tumor-specific antigens, expanding the modified cells, and reintroducing them into the patient after lymphodepleting preconditioning to enhance tumor targeting (Fig 1).^8-13^

Originally developed and approved for hematologic malignancies, CAR-T cell therapy has shown remarkable success in treating diseases such as ALL and diffuse large B-cell lymphoma.^14,15^ This success has spurred significant interest in adapting CAR-T cell therapy for solid tumors, including CRC.^16^

A CAR consists of an extracellular single-chain variable fragment (scFv) domain that binds antigens, a hinge for flexibility, a transmembrane domain to anchor it to the T cell, and intracellular signaling domains for T-cell activation.^17,18^ First-generation CARs use CD3ζ for signaling, while second- and third-generation CARs include costimulatory domains (ie, CD28 or 4-1BB) to enhance T-cell activity and persistence, making them potent tools in cancer immunotherapy.^19,20^

This review aims to present a comprehensive overview of CAR-T cell therapy in CRC, focusing on emerging therapeutic targets, key clinical trials, and challenges. It critically assesses recent advancements and explores the potential of CAR-T cell therapy to alter the therapeutic paradigm for CRC.

## ADVANCEMENTS IN ANTIGEN SELECTION FOR CRC CAR-T CELL THERAPIES

Introducing specific targets for CAR-T cell therapy in CRC is essential for enhancing the efficacy and specificity of the treatment. These targets are carefully selected based on their expression on CRC cells and minimal presence on normal tissues to reduce off-tumor toxicity.^21^ Here, we discuss several key targets for CAR-T cell therapy in CRC (Table 1).

**TABLE 1.** CRC Key Targets for CAR-T Cell Therapy and Their Associated Clinical Trials

Target	Drug Name	Country	Trial Description	Reference No. (ClinicalTrials.gov identifier)
CEA	NA	China	Phase I escalating-dose trial in metastatic CRC	Zhang et al^22^ (NCT02349724)
GUCY2C	NA	China	Phase I escalating-dose trial of GUCY2C-targeted CAR-T therapy in metastatic CRC	Zhang et al^23^ (ChiCTR2100044831)
GUCY2C	IM96	China	GUCY2C-targeted CAR-T tested in advanced CRC	Qi et al^24^ (NCT05287165)
GUCY2C	GCC19CART	China	Phase I escalating-dose trial targeting GUCY2C in relapsed or refractory metastatic CRC	Xiao et al,^25^ Chen et al^26^ (ChiCTR2000040645)
GUCY2C	GCC19CART	The United States	Phase I dose escalation study, targeting GUCY2C in refractory metastatic CRC	Keenan et al,^27^ Schlechter et al^28^ (NCT05319314)
LGR5	CNA3103	Australia	Phase I/IIa, first-in-human study targeting LGR5 in metastatic CRC	Desai et al^29^ (NCT05759728)
NKG2D	CYAD-101	Belgium	Non-gene edited allogeneic CAR-T in metastatic CRC (alloSHRINK trial)	Prenen et al^30^ (NCT03692429)
EpCAM	IMC001	China	EpCAM CAR-T therapy in advanced GI cancers	Luo et al^31^ (NCT05028933)
CDH17	CHM-2101	The United States	Phase I/II CAR-T therapy targeting CDH17 in advanced colorectal, gastric, and neuroendocrine cancers	NCT06055439
MUC1-C	P-MUC1C-ALLO1	The United States	Phase I allogeneic CAR-T targeting MUC1-C epitope in epithelial cancers	Henry et al^32^ (NCT05239143)
CEA	A2B530	The United States	Logic-gated CAR-T targeting CEA in CRC (EVEREST-1 trial)	Molina et al^33^ (NCT05736731)
MSLN	A2B694	The United States	Logic-gated CAR-T targeting MSLN in solid tumors (EVEREST-2 trial)	Punekar et al^34^ (NCT06051695)
NKG2D	CYAD-101 + pembrolizumab	The United States	Allogeneic CAR T-cells CYAD-101 and pembrolizumab in metastatic CRC (KEYNOTE-B79 trial)	Kim et al^35^ (NCT04991948)

Abbreviations: CAR-T, chimeric antigen receptor-T; CDH17, cadherin 17; CEA, carcinoembryonic antigen; CRC, colorectal cancer; EpCAM, epithelial cell adhesion molecule; GUCY2C, guanylyl cyclase C; LGR5, leucine-rich repeat-containing G protein–coupled receptor 5; MSLN, mesothelin; NA, not available; NKG2D, natural killer group 2 member D.

### Carcinoembryonic Antigen

Carcinoembryonic antigen (CEA) is a well-established tumor marker frequently overexpressed in CRC and other gastrointestinal malignancies.^36^ In a phase I clinical trial, the safety, tolerability, and preliminary efficacy of CAR-T cell therapy targeting CEA were assessed in 10 patients with metastatic CRC.^22^ This study used escalating doses of CEA-targeted CAR-T cells. The treatment was generally well tolerated, with seven of 10 patients experiencing no severe adverse effects. However, three patients developed cytokine release syndrome (CRS), with two cases classified as mild to moderate (grade 1-2) and one case as severe (grade 3). Regarding clinical benefit, seven of the 10 patients who had previously experienced progressive disease achieved stable disease (SD) after CAR-T cell therapy. Notably, two patients maintained SD for over 30 weeks, while two others showed tumor reduction based on positron emission tomography-computed tomography (PET-CT) and magnetic resonance imaging analysis, respectively. Additionally, a decline in serum CEA levels was observed in most patients, even during long-term follow-up.^22^ In a related preclinical study, the intraperitoneal (IP) administration of CEA-targeted CAR-T cells was investigated for treating peritoneal carcinomatosis originating from CRC.^37^ The study involved 24 mice, divided into three groups of 8: a control group, a group receiving intravenous (IV) CAR-T cell administration, and a group receiving IP CAR-T cell administration. Results indicated that IP delivery of CAR-T cells was significantly more effective in targeting and eliminating cancer cells compared with IV administration and the control group. Mice receiving IP administration demonstrated better tumor reduction, and high-dose IP CAR-T administration was associated with a more powerful response. Bioluminescent imaging showed no detectable tumor by day 13 in the high-dose IP group, and this response persisted for 76 days after treatment, while 25% of the IV group relapsed by day 27.

Additionally, survival analysis revealed that 75% of mice receiving high or low doses of IP CAR-T cells survived with no tumor burden by day 34, which outperformed the IV group, where some relapse was noted. In extraperitoneal metastases, IP administration conferred durable antitumor activity and eliminated peritoneal implants as well as cleared abdominal solid tumors, making it a more effective route than IV administration.^37^

### Guanylyl Cyclase C

Guanylyl cyclase C (GUCY2C or GCC) is a receptor primarily expressed in intestinal cells and is highly retained in metastatic CRC cells, making it a highly specific target for CAR-T cell therapy in CRC because of its limited expression in other tissues.^38^ Preclinical studies have demonstrated the efficacy of GUCY2C-targeted CAR-T cells in CRC models. These CAR-T cells showed antigen-dependent activation, effectively killing GUCY2C-expressing cancer cells in vitro and providing long-term protection in mouse models against lung metastases of CRC.^39^ Building on these promising preclinical results, a novel GUCY2C-targeted CAR-T cell therapy was tested in an open-label, single-arm trial involving 13 patients with advanced metastatic CRC whose previous treatments were unsuccessful. After lymphodepletion, the CAR-T cells were infused, yielding an overall response rate (ORR) of 60% and a disease control rate (DCR) of 80%. Adverse events were generally manageable, with mild to moderate diarrhea and CRS being the most common, all of which resolved without serious complications.^23^ Further advancing the clinical application of this approach, a phase I trial of GUCY2C-targeted CAR-T cell therapy (IM96) conducted by ImmunoChina evaluated its safety and efficacy in 20 patients with metastatic CRC, including those with liver metastasis, whose disease progressed after at least three previous lines of therapy. Administered in a dose-escalation format, the therapy was generally well tolerated, with no dose-limiting toxicity or maximum tolerated dose reached. The most common side effects included mild to moderate CRS, rash, diarrhea, and oral mucositis, with only one patient experiencing significant neurotoxicity. Among the 19 evaluated patients, the ORR was 26.3%, with a DCR of 73.7%. Notably, in the dose level 3 group, the ORR was 40%, with a median progression-free survival (PFS) of 7 months and a median duration of response of 10 months. Importantly, no patients with an objective response showed disease progression within the first 6 months, indicating durable efficacy and an acceptable safety profile for IM96.^24^

GCC19CART targets GUCY2C and is designed to enhance the proliferation and activation of CAR-T cells by pairing solid tumor-targeting CAR-T cells with CD19-targeting CAR-T cells. A phase I trial is being conducted at the Jilin University, China (ChiCTR2000040645), to assess the safety and preliminary efficacy of GCC19CART in patients with relapsed or refractory metastatic CRC whose two previous lines of systemic therapy were ineffective. Twenty-one patients were enrolled across two dose levels (1 × 10^6^ or 2 × 10^6^ CAR-T cells/kg). The most common adverse events were CRS, diarrhea, and immune effector cell–associated neurotoxicity syndrome (ICANS). These side effects were generally well managed with standard care. ORR of 15.4% and 50% were observed for dose level 1 (DL1) and dose level 2 (DL2), respectively. Preliminary data from this ongoing trial suggest that GCC19CART has dose-dependent clinical activity and an acceptable safety profile in treating relapsed or refractory metastatic CRC.^25^ Recent published data from eight patients treated with a low dose of GCC19CART and seven patients treated with a high dose indicate an ORR of 40%, with six patients showing partial responses (PRs) and an additional five achieving SD. Higher doses were associated with improved PFS, with a median of 6.0 months in the high-dose group compared with 1.9 months in the low-dose group. The median overall survival at the data cutoff was 22.8 months.^26^

Complementing this trial, preliminary data from a US-based study (ClinicalTrials.gov identifier: NCT05319314) on GCC19CART have been published, involving five patients: four treated at DL1 and one at DL2. The study showed a 50% ORR (2/4) at DL1 on the basis of an independent review. Adverse events, including CRS (grade 1 and 2) and ICANS (grade 2), were consistent with earlier findings and resolved with appropriate management. Importantly, two subjects achieved PRs, with an additional patient demonstrating partial metabolic response on PET-CT with SD. The ORR was 25%, with one confirmed PR and three instances of SD as the best observed outcomes. The median PFS in the DL1 group was 3.8 months. These findings highlight the acceptable safety profile of GCC19CART in treating refractory metastatic CRC and underscore the potential for further exploration at higher doses as the trial continues.^27,28^

### Leucine-Rich Repeat-Containing G Protein–Coupled Receptor 5

Leucine-rich repeat-containing G protein–coupled receptor 5 (LGR5) is frequently overexpressed in CRC and serves as a marker for cancer stem cells, which are believed to drive tumor initiation, progression, metastasis, and recurrence.^40,41^ LGR5 is associated with poor survival and response to therapy in metastatic CRC, making it an attractive target for CAR-T cell therapy.^42^ CNA3103 is a first-in-class cell product where autologous T cells are engineered to express a CAR that targets LGR5. The CAR in CNA3103 developed by Carina Biotech includes the antigen-binding domain from BNC101, a humanized monoclonal antibody against LGR5, previously shown to be safe and well tolerated in phase I trials without off-target binding. Up to 44 participants are going to be enrolled across two phases: 24 in phase I and 20 in phase IIa. The trial involves four different dose levels of CNA3103. The study is structured as a first-in-human, multicenter, open-label, phase I/IIa dose-escalation and expansion trial, aiming to assess the safety and overall response to CNA3103 in patients with metastatic CRC.^29^

### Natural Killer Group 2 Member D Ligands

Natural killer group 2 member D ligands are stress-induced molecules expressed on the surface of tumor cells, including CRC.^43^ Targeting them with CAR-T cells can enhance the immune response against CRC. CYAD-101, developed by Celyad Oncology, is an allogeneic CAR-T cell therapy derived from healthy donors, offering a scalable off-the-shelf option. This approach allows for faster production compared with autologous CAR-T cells, which are derived from the patient's own cells.^30^

### Mesothelin

Mesothelin (MSLN) is a cell surface protein overexpressed in several cancers, including CRC, and is associated with tumor aggressiveness and poor prognosis.^44-46^ Histopathologic staining reveals MSLN expression in 48%-61% of CRC cases.^47,48^ CAR-T cells targeting MSLN can exploit its high expression on CRC cells to induce a potent antitumor response.^49^ In a preclinical study, the significant antitumor activity of third-generation MSLN-CAR-T cells was evaluated. The efficacy of MSLN-CAR-T cells was assessed using both cell line–derived xenografts (CDX) and patient-derived xenografts (PDX). In the CRC CDX model (HCT116 cell line), mice treated with MSLN-CAR-T cells (n = 4) showed significantly smaller tumors by day 24 compared with control groups, with no notable changes in body weight, indicating effective tumor suppression. Similarly, in the PDX model, MSLN-CAR-T cells demonstrated strong antitumor effects, even in larger tumors, further supporting their potential as a therapeutic strategy in MSLN-positive CRC.^50,51^

SynKIR-110, developed by Verismo Therapeutics, is another innovative CAR-T cell therapy targeting MSLN-expressing tumors. Using the KIR-CAR platform, this therapy enhances the persistence and efficacy of CAR-T cells in solid tumors. SynKIR-110 has been granted Orphan Drug Designation by US Food and Drug Administration (FDA) and is undergoing clinical trials primarily for mesothelioma and other MSLN-expressing cancers. Although not yet tested specifically in CRC, its potential application could be significant, given MSLN's expression in various solid tumors.^52^

In recent clinical studies, MSLN-targeted therapies have shown promise in treating solid tumors but have raised safety concerns, particularly with respect to lung toxicity. In a phase I/II trial evaluating gavocabtagene autoleucel, a T-cell receptor (TCR) fusion construct targeting MSLN, patients with MSLN-expressing solid tumors, including mesothelioma and ovarian cancer, demonstrated encouraging antitumor responses. However, dose-limiting toxicities, such as grade 3 pneumonitis and fatal bronchoalveolar hemorrhage, were observed at higher doses. Approximately 16% of patients experienced severe lung-related adverse events, leading to a recommendation for careful dosing to reduce risks.^53^ Severe pulmonary toxicity was also reported in a phase I dose-escalation trial of the MSLN-targeted CAR-T cell therapy (M5 huCART-meso). In this trial, two patients in the high-dose cohort developed severe pulmonary complications shortly after infusion, including progressive hypoxemia and extensive lung inflammation. Autopsies indicated that CAR T cells had accumulated within the lung tissue, particularly in benign pulmonary epithelial cells. The observed pulmonary dysfunction is believed to result from the local reactivity of these highly active huCART-meso cells against lung epithelial cells expressing low levels of MSLN. These results suggest that future trials may need to carefully consider dosing strategies and patient selection, particularly for individuals with preexisting lung conditions, to mitigate the risk of such adverse effects.^54^

### Epithelial Cell Adhesion Molecule

Epithelial cell adhesion molecule (EpCAM) is a transmembrane glycoprotein highly expressed on the surface of CRC cells and plays a role in cell adhesion, proliferation, and differentiation.^55^ An EpCAM-targeted CAR-T cell therapy evaluates safety and efficacy of IMC001 developed by ImmunoFoco in patients with advanced gastrointestinal cancers. The trial involved 12 patients with EpCAM-positive cancers, including six patients with colorectal cancer and six patients with gastric cancer. No dose-limiting toxicities were observed during the 4-week follow-up. All patients experienced hematologic toxicity, but these side effects were manageable. One patient developed grade-3 immune hepatitis and required extended hospitalization. Robust CAR-T cell engraftment and prolonged control of circulating tumor cells were seen in all patients, with notable increases in immune markers including interleukin (IL)-6, IP-10, IFN-γ, and IL-15. In an interim analysis of six patients with gastric cancer, two achieved PR and three remained in SD. The first PR patient received a second IMC001 infusion and survived over 60 weeks. The second PR patient successfully underwent gastrectomy 28 weeks after the IMC001 infusion.^31,56^

### Claudin 18.2

Claudin 18.2 (CLDN18.2) is a protein that belongs to the claudin family, which is integral in maintaining tight junctions between cells in epithelial tissues. CLDN18.2 is a specific isoform of claudin 18, primarily expressed in the gastric mucosa.^57^ CT041 is a CAR-T cell therapy developed by CARsgen Therapeutics, targeting CLDN18.2. CT041 uses a CAR structure with a humanized scFv specific to CLDN18.2, a CD8α hinge region, and a CD28 costimulatory domain, enhancing its specificity and efficacy.^58,59^ Although primarily tested in advanced gastric and pancreatic cancers, CT041's success in targeting gastrointestinal tumors suggests potential applicability in CRC.

### Cadherin 17

Cadherin 17 (CDH17), involved in cell-cell adhesion, is overexpressed in gastrointestinal cancers, including CRC.^60,61^ The study by Feng et al demonstrated that CDH17-targeted CAR-T cells could effectively eliminate CRC cells in mouse models without damaging normal tissues.^62^ CHM-2101, an autologous CDH17 CAR-T cell therapy developed by Chimeric Therapeutics, is currently under evaluation in a phase I/II open-label study. This trial focuses on patients with advanced gastrointestinal cancers, specifically those who have relapsed or are refractory to at least one standard treatment regimen in the metastatic or locally advanced setting (ClinicalTrials.gov identifier: NCT06055439).

Similarly, human epidermal growth factor receptor 2 (HER2), another target, is associated with tumor progression in a subset of CRCs. In patient-derived tumor xenografts, HER2 CAR-T cells effectively inhibited CRC progression and demonstrated potent immunotherapeutic effects against metastatic CRC.^63^ In addition, tumor microenvironment (TME)–associated markers could be alternatives for CAR-T cell therapy in CRCs. Fibroblast activation protein (FAP) is highly overexpressed in cancer-associated fibroblasts and is linked to poor prognosis by promoting an immunosuppressive microenvironment in various tumor types.^64^ Recent advances in FAP-targeting immunotherapies, including FAP-specific CAR-T cells, are being investigated in CRC mouse models.^65,66^

## OBSTACLES TO EFFECTIVE CAR-T CELL THERAPY IN CRC

CAR-T cell therapy for CRC faces numerous significant challenges that hinder its effectiveness and safety. One of the primary challenges is the immunosuppressive TME inherent to solid tumors such as CRC.^67-69^ The CRC microenvironment contains various immunosuppressive cells, such as regulatory T cells, myeloid-derived suppressor cells, and tumor-associated macrophages, which can inhibit CAR-T cell function and proliferation.^70,71^ Additionally, CRC tumors often secrete immunosuppressive cytokines such as transforming growth factor-β and IL-10, further dampening the immune response and reducing the effectiveness of CAR-T cell therapy.^72,73^

Another major obstacle is the heterogeneous nature of CRC tumors. CRC cells can exhibit significant genetic and phenotypic variability, leading to the expression of different antigens within the same tumor or between primary and metastatic sites.^74,75^ This heterogeneity makes it difficult for CAR-T cells, which were traditionally engineered to target a single antigen, to effectively eliminate all cancerous cells.^76^ The risk of antigen loss or mutation can result in tumor escape variants, where cancer cells that no longer express the target antigen adapt and spread, causing a relapse.^77-79^

Pharmacokinetic challenges also play a crucial role in the efficacy of CAR-T cell therapy for CRC. CAR-T cells must survive, proliferate, and maintain their functionality over time within the patient's body to exert their antitumor effects. However, in the hostile environment of CRC, CAR-T cells often exhibit reduced persistence and activity, limiting their therapeutic potential.^80^

Moreover, the physical barriers posed by solid tumors, such as the dense extracellular matrix (ECM) in CRC, impede the trafficking and infiltration of CAR-T cells to the tumor site.^81,82^ This limited infiltration reduces the number of CAR-T cells that can reach and attack the cancer cells, significantly affecting the treatment's overall effectiveness.^81^

**FIG 2.**
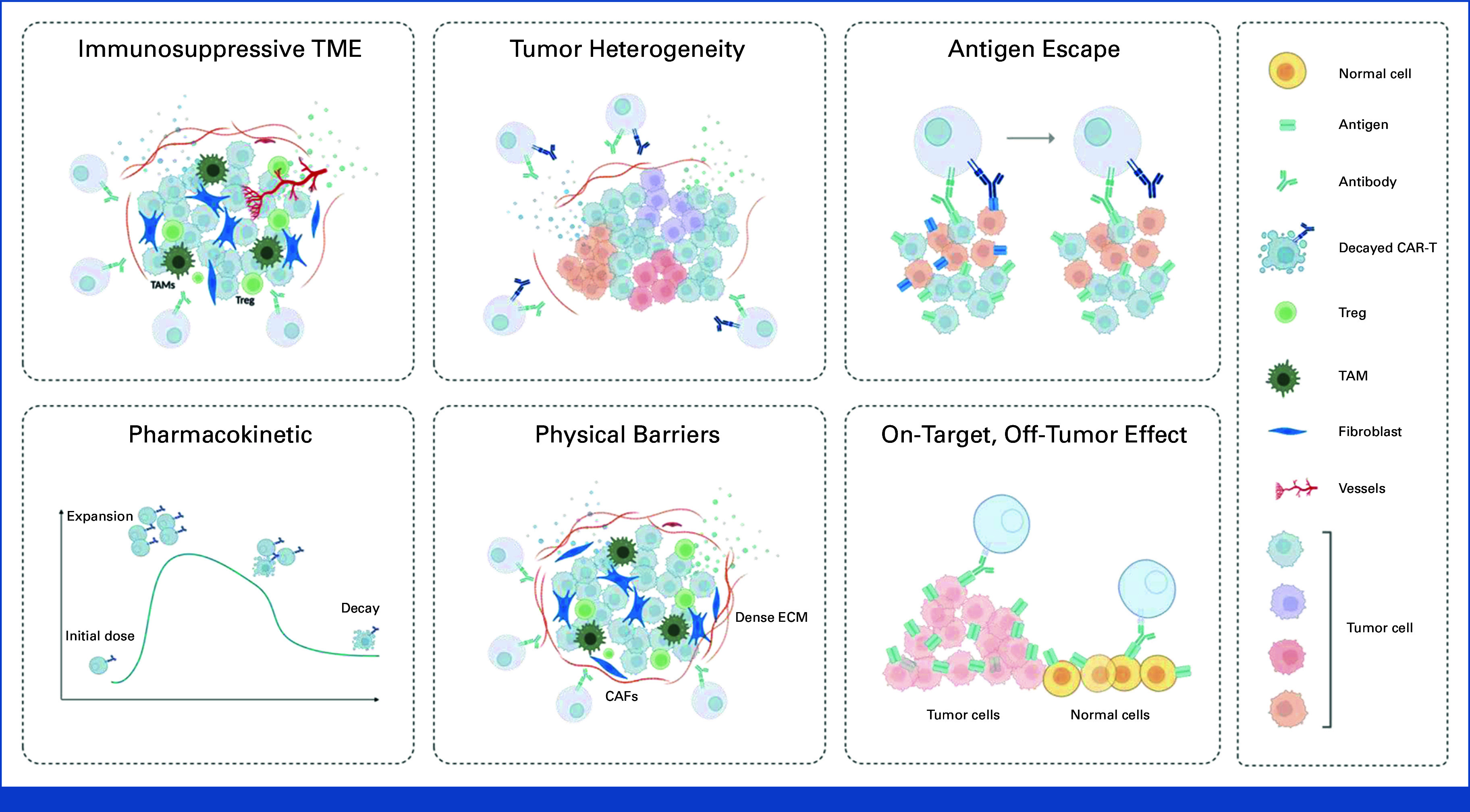
Barriers to effective CAR-T cell therapy in CRC. This figure outlines several key challenges impeding the efficacy of CAR-T cells in solid tumors such as CRC. (Top left) Immunosuppressive TME: the CRC microenvironment is rich in immunosuppressive cells, such as TAMs, Tregs, and MDSCs, which inhibit CAR-T cell function. (Top middle) Tumor heterogeneity: CRC tumors exhibit significant intratumoral heterogeneity, making it difficult for CAR-T cells to target all malignant clones effectively, as different subclones may express varying levels of the target antigen. (Top right) Antigen escape: tumor cells can downregulate or lose the expression of the targeted antigen, resulting in antigen escape, where CAR-T cells are no longer able to recognize and attack tumor cells. (Bottom left) Pharmacokinetics: CAR-T cell persistence and functional efficacy are influenced by pharmacokinetics, where an initial expansion phase may be followed by a decline in cell numbers and activity, limiting sustained therapeutic impact. (Bottom middle) Physical barriers: dense ECM and CAFs in the CRC tumor stroma create physical barriers that impede CAR-T cell infiltration and cytotoxic function within the tumor. (Bottom right) On-target, off-tumor effect: because of the expression of shared antigens between tumor and healthy tissues, CAR-T cells may inadvertently target normal cells, leading to off-tumor toxicity, which is a significant concern in the treatment of CRC. CAFs, cancer-associated fibroblasts; CAR-T, chimeric antigen receptor-T; CRC, colorectal cancer; ECM, extracellular matrix; MDSCs, myeloid-derived suppressor cells; TAMs, tumor-associated macrophages; TME, tumor microenvironment; Tregs, regulatory T cells.

On-target, off-tumor effects and toxicity are additional critical concerns.^83^ CAR-T cells designed to target antigens expressed on CRC cells may also recognize these antigens on normal tissues, leading to unintended damage and severe side effects.^84^ This off-tumor toxicity can cause significant morbidity and requires careful selection of target antigens that are specific to cancer cells while sparing normal tissues (Fig 2).^83^

## STRATEGIES TO BOOST CAR-T CELL EFFICACY IN CRC

**FIG 3.**
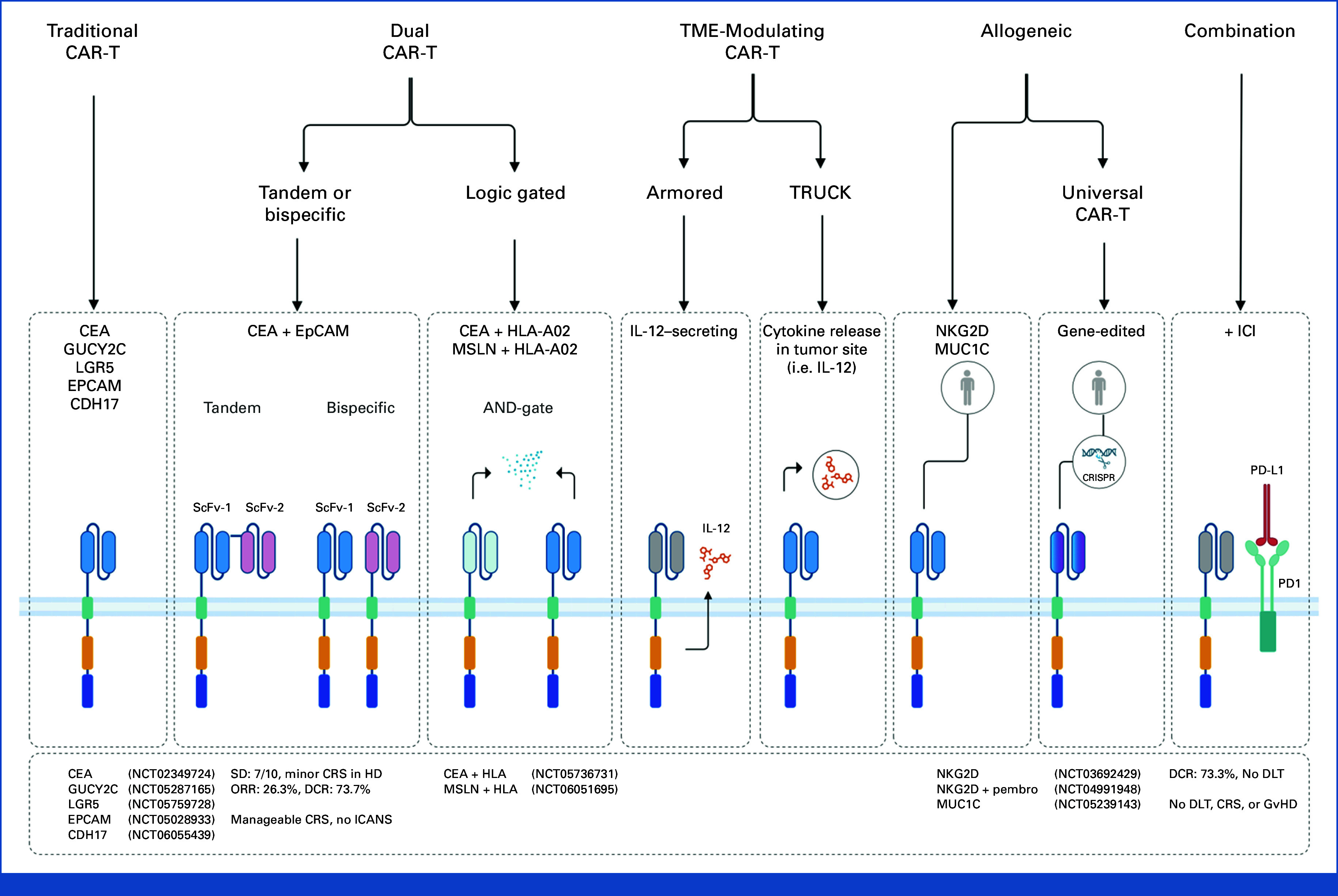
Advancements in CAR-T cell therapies for CRC. This figure illustrates the various innovative CAR-T cell designs aimed at improving efficacy, specificity, and safety in the treatment of CRC. The diagram depicts several generations of CAR-T cell modifications. (1) Traditional CAR-T cells are engineered to target a single antigen on tumor cells. Although effective in hematologic malignancies, their efficacy in solid tumors is limited by antigen heterogeneity and the immunosuppressive TME. (2) Dual CAR-T cells use two distinct strategies. Tandem or bispecific CAR-T cells recognize two different tumor antigens simultaneously, enhancing their tumor specificity and reducing the chances of antigen escape. Logic-gated CAR-T cells use AND or OR gates, requiring multiple conditions (eg, the presence of two antigens) to trigger their activation, reducing the risk of off-tumor activity. (3) TME-modulating CAR-T cells include armored CAR-T cells, which secrete immune-stimulatory cytokines (eg, IL-12) to enhance their own function and reshape the TME to be more immunologically favorable. TRUCK CAR-T cells deliver cytokines directly into the TME, not only attacking the tumor but also stimulating a broader immune response within the hostile tumor environment. (4) Allogeneic CAR-T cells are derived from healthy donors, allowing for an off-the-shelf CAR-T cell product. Universal CAR-T cells within this group are engineered to avoid immune rejection and GvHD, which can occur when using donor-derived T cells. (5) Combination CAR-T cells integrate other approaches such as ICI combined with CAR-T cell therapy. Relevant clinical data, such as ORR and DCR, have been added where available, with NCT numbers included for reference to trials still lacking published results. CAR-T, chimeric antigen receptor-T; CDH17, cadherin 17; CEA, carcinoembryonic antigen; CRC, colorectal cancer; CRS, cytokine release syndrome; DCR, disease control rate; DLT, dose-limiting toxicity; EpCAM, epithelial cell adhesion molecule; GUCY2C, guanylyl cyclase C; GvHD, graft-versus-host disease; HD, high dose; HLA, human leukocyte antigen; ICANS, immune effector cell–associated neurotoxicity syndrome; ICI, immune checkpoint inhibitor; IL, interleukin; LGR5, leucine-rich repeat-containing G protein–coupled receptor 5; MSLN, mesothelin; NCT, National Clinical Trial; NKG2D, natural killer group 2 member D; ORR, overall response rate; scFv, single-chain variable fragment; SD, stable disease; TME, tumor microenvironment; TRUCK, T cells redirected for universal cytokine killing.

Various innovative approaches are being investigated to enhance the proliferation, activation, and persistence of CAR-T cells within the challenging TME of CRC.^85^ Traditional CAR-T cells rely on a single target antigen for activation, which can limit their effectiveness in the complex and heterogeneous TME.^19^ To address these challenges, various advanced CAR-T cell designs have emerged (Fig 3).

### Modulating the Immunosuppressive TME

TME-modulating CAR-T cells are designed to overcome the immunosuppressive effects of the TME. Armored CAR-T cells secrete cytokines such as IL-12, boosting their own function and promoting an immune-friendly environment within the tumor.^86^ Similarly, T cells redirected for universal cytokine-mediated killing (TRUCK) CAR-T cells also secrete cytokines but are designed to recruit other immune cells to the tumor site, further intensifying the antitumor response.^87,88^

Cytokines such as IL-7, IL-12, IL-15, IL-18, and IL-23 are currently being tested with TRUCKs in preclinical models and early-phase trials. This strategy fosters a proinflammatory environment within tumor tissue, enhancing immune activation and amplifying antitumor effects. However, TRUCKs carry a risk of severe side effects if common γ-chain cytokines are continuously released, as this persistent cytokine presence could lead to neurotoxicity and CRS. Consequently, developing strategies for controlled and selective cytokine release is essential to ensure the safe clinical application of TRUCKs.^89^

In a preclinical study, a novel CAR-T cell therapy targeting glypican-3 (GPC3), a protein highly expressed in hepatocellular carcinoma, was investigated. These GPC3-specific CAR-T cells were enhanced with IL-15 to improve persistence and efficacy in both in vitro and in vivo experiments.^90^ Building on these results, a clinical trial (ClinicalTrials.gov identifier: NCT05103631) is currently evaluating the IL-15–armored GPC3-CAR-T cell therapy in patients with GPC3-positive solid tumors. Preliminary findings from the ongoing phase I trial indicate a favorable safety profile, with a DCR of 66% and an ORR of 33%. Some patients did experience CRS, which was managed using an inducible caspase-9 safety mechanism. These early clinical outcomes suggest the potential efficacy of this therapy and underscore the benefit of the IL-15 component in enhancing CAR-T cell performance against solid tumors.^91,92^

### Reducing Time to Treatment With Off-the-Shelf CAR-T Cell Solutions

Allogeneic CAR-T cells, derived from healthy donors, offer the advantage of faster, off-the-shelf availability compared with autologous CAR-T cells. However, they pose risks such as graft-versus-host disease (GvHD) and immune rejection, which advanced gene-editing techniques aim to mitigate by creating universal CAR-T cells.^93-95^ In CRC, allogeneic CAR-T cells have shown promise in early-phase clinical trials.^32,96^ Notably, P-MUC1C-ALLO1 is an allogeneic CAR-T cell product targeting the MUC1-C epitope, which is overexpressed in many epithelial-derived cancers, including colorectal adenocarcinoma. Early results from a phase I trial indicated that this therapy was well tolerated in patients with advanced epithelial cancers, including CRC, without dose-limiting toxicities or severe immune-related adverse events such as GvHD.^32^ Another approach involves allogeneic anti–protein tyrosine kinase 7 (PTK7) CAR-T cells, which target PTK7, a molecule highly expressed in several solid tumors, including CRC. Preclinical studies and early-phase trials have demonstrated that anti-PTK7 CAR-T cells can effectively reduce tumor growth, highlighting their potential as a viable treatment option.^96^

### Augmenting CAR-T Cell Cytotoxicity and Sustained Persistence in Tumor

In a preclinical study, Zhao et al^97^ introduced a novel armed CEA-targeted CAR-T cell therapy that incorporates a SIRPγ-CD28 chimeric coreceptor, specifically designed to enhance the antitumor activity of CAR-T cells against CRC. The SIRPγ-CD28 coreceptor was included to improve the persistence, proliferation, and cytotoxicity of the CAR-T cells. The study involved in vitro assays and in vivo mouse models to evaluate the efficacy of these modified CAR-T cells. Results demonstrated that the armed CAR-T cells exhibited significantly stronger tumor cell killing compared with traditional CAR-T cells, with enhanced resistance to the inhibitory effects of the TME, such as the presence of immunosuppressive cytokines and hypoxic conditions. The modified CAR-T cells also showed improved persistence within the tumor site, maintaining their activity over a longer period.^97^

### Refining Target Specificity and Minimizing Off-Tumor Toxicity

Dual CAR-T cells can be tandem, bispecific, or logic-gated.^5^ Tandem CAR-T cells are engineered to recognize two different antigens simultaneously, enhancing their targeting capabilities, especially in tumors expressing multiple antigens.^98^ Logic-gated CAR-T cells add another layer of specificity, requiring multiple conditions (such as the presence of specific antigens) before they are activated, reducing the risk of off-target effects.^99^

Logic-gated CAR-T cells enhance the precision and safety of solid tumor treatments by requiring multiple conditions to activate.^100^ Using mechanisms such as AND-gate or NOT-gate, these cells target tumors only when specific antigen combinations are present, reducing off-target effects and increasing treatment specificity.^101,102^

Clinical trials, such as those involving CEA-directed Tmod CAR-T cells, are currently exploring the effectiveness of this technology in treating CRC and other solid tumors. A2B530 is a CAR-T cell therapy developed by A2 Biotherapeutics, specifically designed to target CRC expressing CEA and demonstrating human leukocyte antigen (HLA)-A02 loss of heterozygosity. The ongoing EVEREST-1 trial, a phase I/II study, is evaluating the safety and efficacy of A2B530.^33^

Another related trial is investigating the safety and efficacy of A2B694, a logic-gated Tmod CAR-T cell product, in various solid tumors, including CRC, that express MSLN and have lost HLA-A02. The EVEREST-2 trial is a first-in-human, phase I/II, open-label, nonrandomized study aimed at evaluating A2B694. This is an innovative approach to obtain a safer and more effective CAR-T cell therapy for patients with MSLN-expressing solid tumors, potentially overcoming the challenges of on-target, off-tumor toxicity that have hindered similar therapies in the past.^34^

Another example of logic-gated CAR-T cell therapy is the Dual-RevCAR platform, designed for CRC, which targets CEA and EpCAM. The system uses an AND-gate mechanism, activating CAR-T cells only when both antigens are present, thereby increasing specificity and minimizing off-target effects.^103^

### Synergistic Approaches in CAR-T Cell Therapy

Combinatorial CAR-T cell therapy in CRC pairs CAR-T cells with immune checkpoint inhibitors (ICIs) to enhance efficacy by preventing T-cell exhaustion and counteracting the immune-suppressive TME. This approach boosts CAR-T cell infiltration and precise antitumor response.^104^

A preclinical study by Rafiq et al^105^ investigated a novel approach to enhance the efficacy of CAR-T cell therapy by engineering CAR-T cells to secrete a PD-1–blocking scFv. This modification allows the CAR-T cells to block the PD-1/PD-L1 interaction both autocrine (on the CAR-T cells themselves) and paracrine (on bystander T cells). The localized secretion of the scFv at the tumor site potentially reduces systemic side effects associated with ICIs. In mouse models of PD-L1–positive hematologic and solid tumors, the scFv-secreting CAR-T cells demonstrated enhanced antitumor efficacy compared with conventional CAR-T cells. In addition, a follow-up experiment showed that the PD-1–blocking scFv secreted by antigen-irrelevant CAR-T cells could bind to bystander tumor-specific T cells in vivo, enhancing their antitumor function. This was evident in the survival analysis, where a combination of PD-1–blocking scFv and tumor-specific CAR-T cells resulted in significantly improved survival and a robust antitumor response.^105^

The phase Ib KEYNOTE-B79 trial is assessing the safety and clinical activity of sequential therapy combining CYAD-101 with pembrolizumab, an anti–PD-1 monoclonal antibody. In a previous trial (alloSHRINK), CYAD-101 was administered to 15 patients with progressive metastatic CRC after leucovorin calcium (folinic acid), fluorouracil, and oxaliplatin (FOLFOX) chemotherapy. This trial showed that the treatment was well tolerated, with no cases of GvHD. The DCR was 73.3%, with two patients experiencing PRs and nine patients maintaining SD for a median duration of 4.6 months.^30^ Building on these findings, the phase Ib KEYNOTE-B79 trial involves three infusions of CYAD-101 after FOLFOX chemotherapy, with pembrolizumab administered once every 3 weeks for up to 2 years. The pembrolizumab treatment will begin 3 weeks after the final CYAD-101 infusion to avoid overlapping CAR-T cell toxicities, such as CRS. The trial was initially paused by Celyad Oncology in February 2022 because of two fatalities, and the FDA subsequently placed it on hold. Later, the FDA lifted the clinical hold on the CYAD-101-002 (KEYNOTE-B79) phase Ib trial after Celyad Oncology adjusted the trial's eligibility criteria (Fig 3).^35^

## OPTIMIZING CRC PATIENT SELECTION FOR CAR-T CELL THERAPY

Selecting the most suitable patients with metastatic CRC for CAR-T cell therapy is critical for optimizing outcomes. Several factors, including histopathologic subtypes of liver metastases, tumor-infiltrating lymphocyte (TIL) enrichment, and molecular subtypes, may help guide the identification of candidates for this immunotherapy approach.

Liver metastases in patients with CRC can be categorized into three main histopathologic types: desmoplastic, pushing, and replacement.^106^ Each subtype presents a distinct TME that could influence CAR-T cell infiltration and efficacy. Desmoplastic liver metastases are surrounded by a dense fibrotic stroma,^106,107^ which is hypothesized to act as a physical barrier, potentially preventing CAR-T cells from effectively reaching and destroying tumor cells. Patients with this subtype may be less likely to benefit from CAR-T cell therapy because of poor infiltration. Strategies to modulate ECM stiffness are being explored to potentially enhance the effectiveness of cancer immunotherapy.^82^ By contrast, pushing metastases are characterized by a tumor that presses against the liver parenchyma without significant fibrosis.^106,108^ This lack of a dense stromal barrier might make pushing-type metastases more amenable to CAR-T cell therapy, as the cells may more easily infiltrate and exert their effects. Replacement metastases, where the tumor extensively infiltrates liver tissue, pose intermediate challenges because of the complex microenvironment created by the liver's interaction with the tumor.

The density of TILs in the TME is suggested to be a predictor of response to immunotherapy, as high TIL levels are thought to support CAR-T cell function.^109^ Patients with enriched TIL populations may benefit more from CAR-T cell therapy, while those with low TIL levels might require combination treatments, such as ICIs, to potentially enhance immune cell infiltration and effectiveness.^110,111^

Recent advancements in molecular subtyping, including genomic, epigenomic, and pathway-based classifications, have provided deeper insights into the immune activity and responsiveness of different CRC subtypes to CAR-T cell therapy. Among the four consensus molecular subtypes (CMS),^112^ CMS1 (MSI-immune) appears to be an ideal candidate for CAR-T cell therapy because of its immune-enriched microenvironment, characterized by high TILs and elevated immune checkpoint activity. This immune responsiveness could provide an optimal setting for CAR-T cell function. By contrast, CMS4 (mesenchymal), which is associated with extensive stromal infiltration, fibrosis, and poor immune cell presence,^112^ presents significant challenges for CAR-T cell therapy, making it a less favorable candidate. Enhancer-based epigenomic classification (EpiC) further refines the immunogenic potential of CRC subtypes.^113^ EpiC1, which aligns closely with CMS1,^114^ is proposed to be the best match for CAR-T cell therapy, while EpiC4 may not respond well to CAR-T cell therapies because of its less immune-reactive enhancer landscape. The pathway-derived subtypes (PDS) classification emphasizes key signaling pathways that define tumor behavior and immune interaction.^115^ Of the PDS subtypes, PDS-immune aligns most closely with immune-active tumors,^115^ making it a prime candidate for CAR-T cell therapy. This subtype features enhanced immune infiltration and activation, similar to CMS1, and is hypothesized to be well suited for immune-based therapies such as CAR-T cell therapy.

In conclusion, CAR-T cell therapy represents a transformative approach in CRC treatment, providing targeted and personalized immunotherapeutic options. However, challenges such as the immunosuppressive TME, antigen heterogeneity, and physical barriers continue to limit its clinical success.

Comparing various targets and candidate antigens for CAR-T cell therapy in CRC is essential, particularly given the diversity in cell therapy product designs. This comparison should consider critical factors such as antigen expression profiles, specificity, clinical efficacy, safety data from trials, CAR-T cell engineering features, interactions with the TME, and insights from preclinical investigations. A thorough evaluation of these aspects provides a comprehensive understanding of the therapeutic potential of each target, facilitating the identification of antigens that optimize efficacy while minimizing off-tumor toxicity.

Applying such a comparative framework highlights the promise of targets such as GUCY2C, LGR5, and CDH17 for CAR-T cell therapy in CRC. These antigens demonstrate high tumor specificity, encouraging clinical efficacy, favorable safety profiles, and significant potential for optimization through CAR-T cell engineering. By contrast, targets such as MSLN and CEA, despite their overexpression in CRC, face notable challenges that may limit their clinical utility, primarily because of concerns about off-tumor effects. For instance, MSLN-targeted CAR-T cell therapies have been associated with pulmonary toxicity, likely because of MSLN expression on normal mesothelial cells of the pleura and peritoneum. Similarly, CEA-targeted therapies have shown varying degrees of gastrointestinal toxicity, including severe colitis and enteritis, linked to CEA expression on normal gastrointestinal epithelial cells. Although some studies have reported significant toxicity when using murine TCRs against human CEA,^116^ others have noted an absence of such adverse events using CAR constructs,^22^ indicating that differences in cell therapy product design may significantly influence safety outcomes. This nuanced evaluation underscores the importance of balancing antigen specificity and safety considerations in developing CAR-T cell therapies for CRC.

Selecting the appropriate costimulatory domain is critical for enhancing T-cell function, persistence, and the overall therapeutic efficacy of CAR constructs, particularly in solid tumors such as CRC. The CD28 costimulatory domain is known to facilitate robust initial T-cell activation and proliferation, whereas 4-1BB (CD137) costimulation is associated with enhanced T-cell longevity and sustained antitumor activity, which are attributed to improved mitochondrial biogenesis and metabolic fitness.^117,118^ In the context of solid tumors, where long-term T-cell persistence and resilience against the immunosuppressive TME are crucial, 4-1BB may confer significant advantages by augmenting resistance to inhibitory factors prevalent in CRC. Tailoring the costimulatory domain to address the specific immunologic challenges of the CRC microenvironment can thus potentiate CAR-T cell efficacy and improve patient outcomes. Moreover, exploring novel costimulatory domains such as OX40 (CD134) or incorporating dual costimulatory signals may further optimize T-cell function and persistence, offering promising avenues for enhancing CAR-T cell therapy in CRC.^119,120^

Although CAR-T cell therapies have shown promise in treating solid tumors, including CRC, alternative cell types such as TCR-engineered T cells, γδ T cells, and NK cells are being actively explored to overcome the limitations of CAR-T cells.^121^ For instance, CAR-NK cell therapies are under investigation for gastrointestinal cancers, offering advantages such as a reduced risk of GvHD and lower incidence of CRS.^122^ Similarly, TCR-engineered T cells are being developed to target specific tumor antigens, both on cell surfaces and within intracellular compartments, providing an alternative to CAR-T cell therapies.^123^

Future directions involve refining CAR-T cell designs to improve specificity and safety, including the development of logic-gated CAR-T cells and dual-targeting strategies. Advances in gene editing and allogeneic, off-the-shelf CAR-T cell production hold promise for increasing accessibility and reducing treatment timelines. Through strategic antigen selection, optimization of costimulatory domains, and innovative engineering approaches, CAR-T cell therapy has the potential to significantly improve outcomes for patients with CRC and expand its applicability to a broader range of solid tumors.
